# Supplementary material for: Are carbon emissions trading and green financial instruments synergistic? -Comprehensive quantitative research based on content analysis
Source: PLoS One. 2024 Mar 7;19(3):e0298601. doi: 10.1371/journal.pone.0298601 (PMC10919601; doi:10.1371/journal.pone.0298601)
Supplement: S1 Data — (ZIP) [file pone.0298601.s001.zip › Data/地级市Z_F工作报告.zip!/╦─┤¿/╡┬╤⌠╩╨/└┤╫╘╣1⁄2╓┌║┼í╛┬φ┐╦╩2╛▌═°í┐_2014.docx]

各位代表：

我代表市人民政府，向大会报告工作，请予审查。

2013年工作回顾

过去的一年，面对外部经济环境的严峻挑战和“7·9”特大暴雨洪灾的严重影响，全市人民在省委省政府和市委的坚强领导下，牢牢把握“科学发展、加快发展”的工作指导思想，全面实施全省“三大发展战略”和全市“一三五八”发展战略，深化“四个年”活动，攻坚克难、扎实工作，全市呈现经济稳中向好、民生持续改善、社会和谐稳定的良好局面。

一、全力以赴稳增长调结构，经济保持平稳较快发展

积极应对经济增长下行压力，出台工业、投资、消费稳增长的政策措施，加大结构调整力度，努力实现经济转型发展。全市实现地区生产总值1395.9亿元，增长10%；地方公共财政预算收入80.5亿元，同口径增长14.7%。

产业结构不断优化。工业经济稳中有进。实现规模以上工业增加值705.6亿元，增长11.2%；规模以上工业企业达1190户，净增113户。大力实施国家老工业基地调整改造规划，支柱产业转型步伐加快，预计高新技术产业实现总产值770亿元，增长15%，我市获得中国重大技术装备制造业基地授牌。新增2个省级高新技术产业园区，工业集中度达75%。服务业发展加快。实现增加值362.9亿元，增长9.9%，我市被列为省级现代服务业综合改革试点市。现代农业稳步发展。粮食总产量188万吨，同口径增产1.8万吨；肉类总产量37.3万吨，同口径增长3%。建成现代农业产业基地15个，面积超过29万亩。新增农民合作组织206个。

投资保持较快增长。完成固定资产投资807.4亿元，增长10.5%,137个重点项目全部完成年度投资目标。产业投资持续增加，完成工业投资404.9亿元，增长24.1%，天成不锈钢、博力迅电池等117个重大项目竣工投产；完成服务业投资383.1亿元，凯旋国际上美广场等项目建成开业。制定加快民营经济发展的措施，支持民间资本扩大投资领域，民间投资对投资增长贡献率达70.9%。金融运行平稳，金融机构本外币各项存款余额1800亿元，比年初增加110.8亿元；各项贷款余额999.7亿元，比年初增加137.6亿元。银政企合作不断深化，14家省级银行与我市企业签署了3290亿元的战略合作协议。组建交通建设投资有限公司。成立全市首家农业政策性担保公司。

消费拉动明显增强。推进农村市场体系建设和便利消费进社区、便民服务进家庭工程，组织开展端午龙舟美食节、国际文化美食节等促销活动，城乡市场繁荣活跃，实现社会消费品零售总额467.7亿元，增长14.6%。居民消费不断升级，餐饮业转型步伐加快，文化休闲、网络购物等新型消费业态逐步升温。旅游消费快速增长，新增国家3Ａ级景区1处，成功举办全市第三届旅发大会和第二届全球网络旅游营运商大会，实现旅游总收入92.4亿元，增长35%。

创新能力不断提升。新增国家级企业技术中心1户、省级企业技术中心3户、国家高新技术企业23户，建成省级质检中心1个，12项科技成果获省科技进步奖，新增专利实施项目256项，我市荣获全国科技进步先进市称号。产学研合作不断深化，与中科院成都分院签署院市科技合作协议。“双百”工程扎实推进，引进培育力度不断加大。品牌战略取得新进展，新增中国驰名商标1件、国家地理标志保护产品1个、四川省著名商标8件、四川省名牌产品24个。

二、大力推进城乡统筹，城乡环境持续改善

围绕构建现代城镇体系，加快城乡基础设施建设，提升城镇功能，全市城镇化率达45.9%，提高1.1个百分点。

基础设施建设全面提速。加快构建现代综合交通体系，国道108线示范工程、旌江干线完工通车，中金快速通道中江段主体工程完工，天星干线、绵茂公路一期工程加快建设，配合做好成绵乐城际铁路、成兰铁路、成都二绕等工程建设。完成通乡公路72.1公里、通村公路90.1公里。农田水利建设力度加大，完成重点水利项目投资9.8亿元，八角水库完成施工招标，华强沟水库前期工作有力推进，建成高标准农田12.9万亩，新增有效灌面9.9万亩，治理水土流失面积122平方公里。投运国内首座110千伏移动变电站。

城乡建设力度加大。强化规划引领，完成城市总体规划修编评估报告、新区核心区控制性详规、城市慢行交通等专项规划。中心城区建设步伐加快，实施54个市政基础设施项目，总投资34.14亿元，站前广场、旌南大厦等25个项目竣工，南北汽车站投入运营，东一环路加快建设。创新新区开发建设体制机制，旌东新区起步区、亭江新区中央创新区建设全面启动，37个基础设施项目开工建设。县域城镇建设步伐加快，5个镇被纳入全省百镇试点行动。新农村建设扎实推进，新建182个新村聚居点和19个新农村综合体，建成一批“幸福美丽新村”示范点，广汉兴隆镇被命名为全国一村一品示范村镇。启动智慧城市建设。

生态环境不断改善。强化节能减排，狠抓重点行业污染整治、清洁生产和淘汰落后产能，关停7户小煤矿，预计万元GDP能耗下降4%，主要污染物减排任务全面完成。秸秆禁烧和综合利用工作成效明显，环境质量持续改善。生态修复扎实推进，新增森林面积2.67万亩，完成营造林6.7万亩。生态宜居城市建设成效显著，成功创建国家园林城市和省级文明城市、森林城市、环保模范城市。广汉、旌阳成功创建省级生态县，罗江成功创建省级绿化模范县。

三、深入推进改革开放，发展活力不断增强

适应经济社会发展的新形势，创新体制机制，实施充分开放合作，发展活力和发展动力不断增强。

重点领域改革不断深化。编制完成统筹城乡规划和产业发展总体规划，一体化的梯次规划体系逐步建立。农村综合改革稳步推进，农村土地确权登记工作基本完成，各项配套制度改革步伐加快。户籍制度改革取得突破，城镇落户限制条件全面放开。深化国库集中收付制度改革，全面实现预算单位和资金支付全覆盖。营业税改征增值税试点工作稳步推进。组建政府公共资源交易中心。完成食品药品监督管理体制改革。妥善处理历史遗留问题，市属国企改革稳妥推进。

招商引资成果丰硕。创新投资促进服务方式，积极参与“民企入川”、“央企入川”、“港澳企业四川行”、第十二届世界华商大会和第十四届西博会等投资促进活动，成功引进工业云制造基地、九为蓝谷总部基地等一批重大项目，到位内资638.3亿元、增长23.6%，到位外资2.03亿美元、增长4.9%。

外向型经济发展加快。完成进出口总额33.9亿美元、增长9.7%，其中出口23.5亿美元、增长29%。口岸建设取得突破，海关、出入境商品检验检疫即将开关开检。对外交流合作不断扩大，我市与韩国江陵市缔结为友好城市。对台工作不断加强。

区域合作开启新篇。签订成德同城化框架协议，建立两地联席会议协调机制，全面启动规划、交通、产业等方面的合作，与成都、绵阳合作完成《成德绵区域合作总体规划》，在中江与成都共建成德工业园。对口支援若尔盖县、阿坝县工作深入推进，已开工40个援建项目，完工14个。推动在绵竹与阿坝州共建德阿产业园。对口支援芦山县灾后重建工作扎实开展。

四、全面推进社会建设，人民生活持续改善

以保障和改善民生为重点，加快社会事业发展，不断提高人民群众生活水平。城镇居民人均可支配收入24701元，增长10.4%；农民人均纯收入10094元，增长12.7%。

“十大民生工程”全面完成。各级财政投入民生工程资金48.9亿元，增长11.4%。创业带动就业成效明显，新增城镇就业3.5万人，城镇登记失业率控制在4%以内，藏区“9+3”毕业生初次就业率达100%。义务教育“两免一补”实现城乡全覆盖，资助高中、中职学校家庭困难学生2.62万人，中职学校学生免学费2.89万人，资助普惠性幼儿园家庭经济困难儿童、孤儿、残疾儿童8532人。设立市级财政专项扶贫资金，扶持6.03万农村贫困人口改善生产生活条件。开工保障性安居工程6210套，完成农村危房改造3360户。搬迁安置400户农户地质灾害防灾避险。解决9万农村人口饮水安全问题。

社会保障水平不断提高。社会保险扩面提标，新增城镇职工养老保险参保5.64万人、城乡居民养老保险参保3.18万人、城镇基本医疗保险参保6.86万人，新农合人均筹资标准提高到340元。建立疾病应急救助制度，启动城乡居民大病保险试点。上调全市最低工资标准，企业退休职工养老金、城乡低保标准和农村“五保”供养标准进一步提高。

公共文化覆盖城乡。免费开放图书馆、文化馆、乡镇文化站，建成示范乡镇综合文化站24个。农村广播电视村村通工程、广播电视无线覆盖工程和电影放映工程有序推进。培育具有时代特征的城市精神，确定“古蜀之源、重装之都”为德阳城市主标识。群众文化活动遍布城乡，举办德阳灯会、广汉保保节、绵竹年画节等民俗文化品牌活动。全民健身运动蓬勃开展，第四届环中国国际公路自行车德阳赛段比赛成功举行，新建全民健身苑16个、全民健身路10条。

教育卫生事业加快发展。新建、改建幼儿园46所，学前三年幼儿入园率92.3%。义务教育均衡发展取得新进展，绵竹和广汉义务教育均衡发展通过省级验收，改造农村义务教育薄弱学校262所。普通高中教育、职业教育质量全面提高，建成2所国家级示范性高职学院，3所中职学校成为国家示范建设单位，四川师范大学广汉科技园建成省级大学科技园。医疗卫生体制改革深入推进，政府办基层医疗卫生机构实现基本药物制度全覆盖，全面取消县级公立医院药品加成。基层中医药服务能力明显提高。重大传染病得到有效控制，绵竹、什邡实现血吸虫病传播阻断达标。持续保持低生育水平，人口自然增长率2.15‰。

社会保持和谐稳定。社会治安综合治理整体联动防控体系不断健全，流动人口、特殊人群和虚拟社会管理取得明显成效，群众安全感稳步提高。社区建设扎实推进，完成220个城乡社区规范化、标准化建设，建成社会组织孵化培育基地。加强群众和信访工作，及时预防和化解社会矛盾。强化危化品等重点行业治理，开展食品安全专项整治，安全生产形势持续好转。强化防灾减灾和应急处置，积极参与“4·20”芦山地震抢险救援，成功抗击“7·9”洪灾。国防动员、民兵预备役和“双拥”工作继续加强。

统计调查、档案、民族宗教、住房公积金、老龄、妇女儿童、残疾人、人防、保密、地方志、气象、红十字会等各项工作取得新进展。

各位代表，过去的一年，政府自觉接受市人大及其常委会和市政协的监督，广泛听取各民主党派、工商联及社会各界的意见建议，办理人大代表建议87件，政协委员提案234件。扎实开展法治政府创建工作和“行政执法规范年”活动，建成行政权力依法规范公开运行平台。第九届村（居）委会换届工作有序推进。深化行政审批制度改革，取消、调整164项行政审批项目。创新服务方式，推行“一窗式”投资项目全程代办服务和企业零成本注册等新举措，开通“12345”为民服务热线，行政效能进一步提升。严格落实“八项规定”，狠刹公款消费等不正之风，加强对重点领域、关键环节廉政风险监管，严厉查处违法违纪案件，廉政建设得到加强。深化“作风转变年”工作，深入开展“走基层、建台账、惠民生”活动，加大效能投诉处理力度，行政作风进一步改进。

各位代表，过去一年的成绩来之不易，凝聚着全市人民的智慧和汗水，饱含着社会各界的关心和支持。在此，我代表市人民政府，向市人大代表、政协委员和离退休老同志，向各民主党派、工商联、无党派人士、人民团体和全市人民，向驻地人民解放军、武警官兵和公安干警，向所有关心、支持德阳发展的海内外朋友，表示衷心的感谢并致以崇高的敬意！

在肯定成绩的同时，我们也清醒地看到存在的问题和不足：受宏观经济持续下行和自身经济结构性问题的影响，经济增速有所回落，地区生产总值等指标未实现预期目标；传统产业转型升级任务较重，服务业比重偏低，战略性新兴产业支撑作用不强；统筹城乡发展体制机制不够完善，城乡基础设施和生态环境有待进一步提升；城乡居民持续增收难度加大，保障和改善民生的任务较重；群众利益诉求日趋多样，社会稳定压力依然较重；个别部门和少数公职人员作风不实、服务意识不强，政府公共服务和依法治理能力还需提升。对于这些问题，我们高度重视并下大力气加以解决。

2014年工作安排

今年是贯彻党的十八届三中全会精神的第一年，也是完成“十二五”规划任务的关键之年。当前，我们正处于转型发展的关键时期，既面临发展环境深刻变化的严峻挑战，更面临全面深化改革的重大机遇。我们要认清形势，增强忧患意识，抢抓发展机遇，以更大的决心和更大的力度推动德阳改革发展。

今年政府工作的总体要求是：认真贯彻党的十八大、十八届三中全会、省委十届三次全会精神，全面落实中央和省委经济工作会议部署，用改革统领经济社会发展全局，坚持科学发展、加快发展的工作指导思想，深入实施全省“三大发展战略”和全市“一三五八”发展战略，深化“四个年”活动，加快推进经济结构调整和发展方式转变，统筹“五位一体”建设，切实保障和改善民生，打造质量型增长、内涵式发展新格局，为在全省率先实现次级突破打下坚实基础。

今年经济社会发展主要预期目标：地区生产总值增长10%；规模以上工业增加值增长11%；全社会固定资产投资880亿元；地方公共财政预算收入同口径增长11%；社会消费品零售总额增长13%；城镇居民人均可支配收入增长10%；农民人均纯收入增长11%；城镇登记失业率控制在4%以内；人口自然增长率控制在3.6‰以内；全面完成省下达的节能减排任务。

一、加快转型升级，实现更高质量发展

转型升级事关德阳未来。要大力推进产业结构调整，努力提升德阳产业核心竞争力。

再造工业竞争新优势。坚持改造传统产业与培育新兴产业并重，产业外延增长与内涵发展并重，培育优质增量与优化存量并重，全力推动产业升级。加快老工业基地改造，大力促进传统装备制造业升级换代，着力构建现代装备制造体系。通过行业准入标准、节能减排、环境保护等倒逼机制，促进企业转型发展，推动化工精细化、清洁化。引导食品行业适应消费需求，开发适销对路的新产品。深入推进战略性新兴产业“5+2”工程，力争在通用航空、储能电池、节能环保等领域形成产业集群竞争优势和技术领先优势。狠抓工业项目，完成工业投资430亿元。提升园区承载力，注重节约集约用地和引进项目质量。强力推进广汉建设国家级高新区。加速信息化与工业化深度融合，积极探索云制造模式，鼓励企业采用物联网、3D打印等现代技术手段，提高产品设计开发能力。

加快发展现代服务业。加大对服务业发展的规划和政策引领，扎实开展省级现代服务业综合改革试点。大力发展现代物流、现代金融、科技信息、创意研发等生产性服务业，推动制造企业分离发展现代服务业。结合新型城镇化加快发展社区服务、养老服务、健康医疗等生活性服务业。加快城市商圈、特色商业街发展，抓好黄许物流园等现代物流园区建设。积极发展电子商务、消费金融、信息消费、通用航空服务等新型业态，培育新的消费热点。精心打造六大特色文化旅游品牌，搞好旅游产品开发和营销，提升德阳旅游吸引力。

优化发展现代农业。稳定发展农业生产，抓好现代粮食产业示范基地和现代畜牧业重点县建设，创建一批现代农业万亩亿元示范区、“千斤粮万元钱”基地和畜禽标准化适度规模养殖小区。调整农业产业结构，加快建设一批高端农业产业基地。大力发展“粮经复合”、“种养复合”、“林经复合”和“旅经复合”，促进传统农业向质量效益型、生态观光型农业转变。扶优扶强农业产业化龙头企业，推进农民合作组织规范建设，培育家庭农场、农民职业经理人等新型农业经营主体。深入实施农民收入促进计划，推动农民持续稳定增收。

二、狠抓项目建设，保持投资较快增长

稳增长关键是稳投资，要以项目为支撑加大投资力度，调整优化投资结构，进一步增强投资的拉动作用。

加快重点项目建设。强力推进总投资1512亿元的142个省市重点项目，确保完成年度投资256亿元。加快绵茂公路、天星快速干线等重点交通项目建设，实现广青公路三期、中金快速干线通车，开工成都经济区环线高速公路德阳段，配合做好成都二绕高速公路等工程建设。加强农田水利建设，抓好重点水利工程项目，开工华强沟水库，力争开工石泉水库，加快推进三渔水库、姜家桥水库前期工作。搞好“7·9”洪灾损毁道路、桥梁等工程修复重建。狠抓产业项目开工建设、竣工投产和量产增效。继续抓好电力、通讯等基础设施建设。

强化项目管理。主动对接国家公共投资重点方向和产业政策导向，围绕节能环保、棚户区改造、信息消费、城市基础设施等重点领域，加强项目对接，谋划和储备一批支撑经济社会发展的重大项目，确保投资持续较快增长。完善责任落实、目标倒逼、市场运作等项目推进机制，破解用地、能源、征地拆迁等制约瓶颈，强化以政务服务中心为平台的投资项目全程代办服务，促进投资项目加快建设。

创新投融资机制。坚决破除各种隐性壁垒，引导更多社会资本进入战略性新兴产业、公用事业和民生领域。认真落实银政战略合作协议，鼓励金融机构加大信贷投入，稳步扩大社会融资总量。加强投融资平台建设，综合运用地方政府债券和BT、BOT、TOT等融资方式，支持新型城镇化和基础设施建设。鼓励企业利用债券市场直接融资，推进中小企业“区域集优”直接债务融资。加强企业上市培育，支持企业通过多层次资本市场直接融资。加快推进地方法人金融机构的改革与发展。支持农村信用社、村镇银行、融资性担保公司、融资租赁、风险投资、金融仓储等业务发展。强化金融生态环境建设，依法打击和处置非法集资，加快推进社会信用体系建设。

三、坚持城乡统筹，促进新型城镇化加速提质

抓住新型城镇化发展机遇，大力推进以人为核心的城镇化，着力构建城乡一体化发展新格局。

构建现代城镇体系。加快实施“1+5+X”城镇体系发展规划，大力推进组团城市群建设，力争城镇化率提高1.6个百分点。科学定位县域城市职能，推动县域之间资源整合和分工协作，强化市级在重大规划、基础设施建设、产业项目布局等方面的统筹力度。加强基础设施建设，提高县域中心城市综合承载能力。加快建设一批5万人口以上的区域中心镇，积极引导产业、人口、资源和公共服务集聚。坚持“五位一体”整体推进，高标准建设一批产村一体、功能完善的幸福美丽新村。

全面提升中心城市功能。统筹抓好旧城改造和新区建设，营造高品质的城市生活空间。打好中心城市升级攻坚战，结合城市总体规划、老工业基地调整改造规划，加快实施中心城区更新改造、旧城危房和棚户区改造，促进城市落后工业区、落后商业区、城中村向产城新区和现代化新社区转型。加大道路、电力、防洪排涝等基础设施建设和改造力度，优先发展城市公共交通，实现东一环路建成通车。科学开展城市新区建设，做好新区各种业态的详规和设计。抓好起步区重大基础设施建设，开工漓江路景观中轴线、漓江路大桥等项目，加快牡丹江路、秀山街等续建项目，完成绵远河生态整治等项目建设。强化起步区产业支撑，抓紧布局重大产业项目，努力建成引领区域产业升级的创新核心。加快打造光网城市、无线城市和智慧城市。

提高城市管理水平。创新城市管理体制机制，全面推行网格化城管模式，完善数字化城管系统。深入开展市容市貌整治，深化背街小巷、城乡接合部、农贸市场等重点区域治理，强化车辆乱停乱放、户外广告乱象、占道经营等突出问题整治。抓好环卫基础设施建设，大力推广垃圾分类试点和清洁直运，推进城市垃圾无害化处置。抓住城市文化底蕴、人文特质，布局特色风貌，彰显城市个性，打造一批精品绿地和特色景观。巩固“五城联创”成果，争创国家卫生、文明、环保模范和森林城市，实现城市环境、形象、内涵和品质的全面提升。

四、深化改革创新，增创科学发展体制机制新优势

全面深化改革是时代赋予我们的伟大使命。要坚持问题导向，勇于破解难题，努力释放以改革促发展的强大动力。

创新驱动增强发展活力。围绕创新型城市建设，大力实施“六大科技工程”，加大创新投入，培育创新主体，全方位优化创新环境。鼓励企业建设高水平研发机构，深化与科研机构和大专院校的战略合作，促进核心技术研发能力和科技成果产业化能力双提升。围绕产业需求开展核心关键技术攻关，推动重型燃气轮机、石墨烯、电动汽车、大功率铝空电池、磷酸铁锂材料等关键技术尽快实现产业化。加强科技企业孵化器、产业技术研究院、工程技术中心、产业联盟等创新载体建设。深入实施专利、标准、品牌战略，鼓励企业经营模式创新和品牌营销，着力推动产业向高端延伸。坚持高水平培养和高质量引进并举，集聚一批科技领军人才和创新团队。

深化改革注入发展动力。认真落实中央和省的改革部署，大力推进重点领域和关键环节的改革。支持国有企业股份制改造和建立现代企业制度，大力发展混合所有制经济，积极稳妥推进市属国有企业改革。深化财政体制改革，改进预算管理制度，扩大国有资本经营预算实施范围，提高财政运行绩效，建立健全政府债务风险防控机制。深入推进农村产权制度改革，开展农村产权股份量化工作，健全农村产权流转交易市场，开展农村产权抵押融资。有序推进社会事业、社会治理、生态文明等领域的改革。

激发民营经济活力和创造力。毫不动摇鼓励、支持、引导非公有制经济发展，完善促进民营经济发展的配套政策，让所有企业享有平等权力、平等机会、平等规则。实施民营企业培育成长行动，着力培育一批竞争力强、带动作用大、发展前景广阔的大企业大集团。大力扶持中小微企业特别是科技型中小微企业发展，切实帮助企业解决融资、要素保障等方面的困难。支持民营企业通过股份制改造、技术创新、品牌培育等方式转型升级。加强行业自律，支持行业协会商会充分发挥作用。

五、加快成德同城，建设内陆开放高地

实行更加积极主动的开放战略，推动开放朝着优化结构、拓展深度、提高效益方向转变，培育开放型经济新优势。

推进成德全方位合作。全面落实成德同城化发展框架协议，努力促进两市经济社会更加紧密融合。抓好规划对接，共同编制《成德同城化空间发展规划》和基础设施、产业发展等专项规划。建立近、中、远期重点合作项目库，启动一批重点合作项目和标志性工程。开展产业协作，加快成德合作工业园建设。全面推动旅游共享、环保联动、金融合作，积极开展教育、就业、文化等社会公共事业领域的合作。加快高效便捷的战略通道建设，谋划和布局对接成都的轨道通道。

提高招商引资实效。围绕供应链、产业链、价值链的高端领域招大引强，确保到位内资650亿元，利用外资2.05亿美元。主动对接世界500强和国内优势企业，全力引进战略性新兴产业、先进制造业和现代服务业领域的重大项目，加大产业链薄弱环节以及龙头企业、关键配套企业的招引力度，积极吸引优势企业和机构在德阳设立地区总部、研发中心和结算中心。强化签约项目跟踪落实，提供最优质的环境和最高效的服务，促进项目尽快落地。

大力发展外向型经济。推动外资、外贸、外经提质发展，完成进出口总额35亿美元。加大出口企业培育力度，创新加工贸易模式，大力发展服务贸易，逐步形成以技术、品牌、质量、服务为核心的出口竞争新优势。加快“走出去”步伐，支持企业发挥自身优势到境外开展投资合作，建立营销网络和生产基地。抓好海关、出入境商品检验检疫局开关开检和综合保税区申报筹建工作。充分发挥外事、侨务、对台在扩大开放中的积极作用。

深化对口援建和区域合作。着力民生援建、产业援建和智力援建，抓好对口援建芦山县、阿坝县和若尔盖县工作，强化与受援县在特色农产品、畜牧业、旅游业等方面的合作。高标准建设德阿产业园，支持罗江与绵阳涪城区开展工业园区合作。继续深化与北京、江苏援建省市的合作，加强与长三角、珠三角、环渤海等区域的合作，扩大与港澳台地区投资和经贸合作，拓展与友好城市的交流合作，全面提升城市国际化水平。

六、建设生态文明，打造美丽德阳

良好的生态环境是最普惠的民生福祉。要把生态文明建设摆在更加突出的位置，推动实现更长远、更持续、更高质量的发展。

强化环境整治。狠抓大气环境综合治理，加大餐饮业油烟、建筑工地扬尘、工业废气、秸秆焚烧等污染治理力度，实施PM2.5监测和公布。加强河流治理，积极开展沱江流域德阳段污染综合防治，抓好旌湖水环境整治，加快污水处理设施及配套管网建设，加强饮用水水源地保护。强化污染源综合防治，全面开展机动车环保标志核发工作，加大对重点污染行业及畜禽养殖污染整治力度，推动实施生活垃圾焚烧发电工作。

保护自然生态。加强自然生态系统保护和修复，深入实施退耕还林、天然林保护和林业产业“2112”工程，加强自然保护区和森林公园建设，巩固生态植被恢复成果。开展河道生态建设，加强沱江上游源头生态环境保护。搞好重大地质灾害隐患治理和水土保持工作。深化生态细胞创建工作，推进生态市建设。

促进节能减排。强化企业节能降耗技术改造，加快淘汰落后产能，积极推进公共建筑、工业、道路照明等重点领域合同能源管理，支持节能低碳产业和可再生能源发展。狠抓污染减排，严格总量控制，扎实推进主要污染物总量指标有偿使用工作。加大耕地保护力度，强化高耗水行业节水技术应用，加强循环经济技术研发推广，提高土地、水、电、矿产等资源利用效益。抓好国家节能减排财政综合评审示范市和再生资源回收利用试点市申报工作，深化节水型社会示范区建设。

七、加强社会建设，保障和改善民生

以促进社会公平正义、增进人民福祉为出发点和落脚点，大力实施民生工程，实现发展成果更多更公平惠及全市人民。

全面提升就业社保水平。狠抓就业促进，强化职业技能培训，努力促进高校毕业生、城镇就业困难群众、退役军人、被征地农民和新生代农民工的就业，确保城镇新增就业3.4万人。统筹推进城乡社保体系建设，持续提高城乡居民养老保险覆盖面和基本医疗保险参保率，推进社会保障由制度全覆盖向人员全覆盖转变。加快建设社会养老服务体系。完善社会救助体系。大力发展慈善事业和残疾人事业。加强农村危房改造，开工建设保障性安居工程8648套。抓好扶贫和移民后扶工作，扶持4.62万农村贫困人口改善生产生活条件，提高发展能力。

推动文化繁荣发展。践行社会主义核心价值观，弘扬感恩奋进精神，倡导健康文明的社会风气，提高城市公共文明指数。加快推进重点文化基础设施建设，高水平建设一批公共文化设施。丰富群众文化生活，加强文化精品创作，打造一批有影响力、竞争力、特色鲜明的文化品牌。大力发展现代文化产业，推动演艺娱乐业、动漫游戏业、现代传媒业加快发展，提升三星堆文化、三国文化、德孝文化、红色文化等文化品牌。深入开展全民健身活动，强化公共体育设施建设，提高竞技体育水平。

提供更好的教育卫生服务。深化教育改革，加快发展学前教育，均衡发展义务教育，提升普通高中教育质量，推动高等教育、职业教育高水平发展。大力推进国家高等职业教育综合改革试验区建设，支持四川工业管理职业学院专升本，力争四川护理职业学院一期工程建成招生。健全全民医保体系，开展城乡居民大病保险、城乡困难群众重特大疾病医疗救助工作。深化基层卫生机构综合改革，抓好公立医院改革，整合区域公共卫生服务资源，支持社会办医，发展中医药事业。搞好重大疾病和地方病防控。稳妥推进生育新政，保持适度生育水平，促进人口长期均衡发展。

创新社会治理。深入推进“平安中国”示范区建设，大力实施主动警务战略，依法防范和打击违法犯罪活动。创新社会矛盾预防化解，完善法律援助制度，建立畅通有序的诉求表达、心理干预、矛盾调处、权益保障机制。激发社会组织活力，培育发展公益慈善类、城乡社区服务类等社会组织。严格落实安全生产“一岗双责”，加强道路交通等重点领域安全整治，积极开展安全社区和企业安全生产标准化建设，坚决遏制重特大安全事故。加强食品药品生产、流通、消费全过程监管，坚决打击假冒伪劣。完善应急预案体系，提高应急管理水平。抓好第三次全国经济普查工作。关注留守妇女、儿童身心健康。做好国防教育、国防动员、民兵预备役和拥军优属工作。

八、加强自身建设，努力建设法治政府和服务型政府

围绕更好发挥政府作用，深化行政管理体制改革，创新行政管理方式，强化政府服务，增强政府公信力和执行力。

转变政府职能。正确处理政府与市场、政府与社会的关系，充分发挥市场在资源配置中的决定性作用，进一步简政放权，最大限度减少政府对微观经济运行的干预。全面梳理政府管理和介入的事务，放开应该由企业和社会组织自我服务、自我管理的事项。强化政府公共服务、市场监管、社会管理、环境保护等职责。引入竞争机制，扩大购买公共服务范围。稳步推进政府机构改革和事业单位分类改革。

推进依法行政。主动接受市人大及其常委会的法律监督、工作监督和市政协的民主监督，自觉接受社会监督和舆论监督，认真办理人大代表建议、政协委员提案。积极推行开放式决策，提高科学决策水平。强化规范性文件备案审查，规范行政执法自由裁量权，加强对行政执法的层级监督。大力推进政务公开，搞好电子政务建设，强化行政效能监察，让权力在阳光下运行。深入开展法治政府创建活动，普遍建立法律顾问制度，运用法治思维和法治方式解决问题、推动工作。

强化政府服务。精简行政审批事项，做好减少和下放审批事项的对接工作，不断深化“两集中、两到位”，积极探索网上联审、容缺预审等方式创新，推行工商注册制度便利化，搭建集中、公开、规范的中介服务平台，提高办事效率。进一步完善目标绩效管理、闭环管理、督查督办机制，大力推行台账工作法。狠刹遇事推诿、办事拖拉等不良风气，严肃问责行政不作为、慢作为、乱作为。

加强廉政建设。健全反腐倡廉制度体系，完善教育、预防、监督和惩治制度，坚持用制度管权管事管人。推行行政权力清单制度，依法公开权力运行流程。强化在财政资金使用、公共资源交易等重点领域的监管，严肃惩处权力寻租、权钱交易等违法违规行为。扎实开展群众路线教育实践活动，健全改进作风常态化机制。严格落实《党政机关厉行节约反对浪费条例》和《党政机关国内公务接待管理规定》，严控“三公”经费支出。加强财政监督和审计监督。

各位代表，时代足音铿锵有力，全面小康催人奋进。让我们紧密团结在以习近平同志为核心的党中央周围，深入贯彻科学发展观，振奋精神、开拓进取、真抓实干，为争当四川科学发展排头兵，加快建设中国西部经济文化强市，在全省率先全面建成小康社会而努力奋斗！
